# *In vitro* protection of biological macromolecules against oxidative stress and *in vivo* toxicity evaluation of *Acacia nilotica* (L.) and ethyl gallate in rats

**DOI:** 10.1186/1472-6882-14-257

**Published:** 2014-07-21

**Authors:** Shalini Mohan, Kalaivani Thiagarajan, Rajasekaran Chandrasekaran, Joseph Arul

**Affiliations:** 1 School of Bio Sciences and Technology, VIT University, Vellore, Tamilnadu 632014, India; 2 Department of Food Science and Nutrition, Laval University, Quebec, QC G1V 0A6, Canada

**Keywords:** *Acacia nilotica* (L.), Ethyl gallate, Antioxidant, Oxidative damage, Toxicity, DNA interaction

## Abstract

**Background:**

Recently, enormous research has been focused on natural bioactive compounds possessing potential antioxidant and anticancer properties using cell lines and animal models. *Acacia nilotica* (L.) is widely distributed in Asia, Africa, Australia and Kenya. The plant is traditionally used to treat mouth, ear and bone cancer. However, reports on *Acacia nilotica* (L.) Wild. Ex. Delile subsp. *indica* (Benth.) Brenan regarding its toxicity profile is limited. Hence in this study, we investigated the antioxidant capacity and acute toxicity of ethyl gallate, a phenolic antioxidant present in the *A. nilotica* (L.) leaf extract.

**Methods:**

The antioxidant activity of ethyl gallate against Fenton’s system (Fe^3+^/H_2_O_2_/ascorbic acid) generated oxidative damage to pBR322 DNA and BSA was investigated. We also studied the interaction of ethyl gallate to CT-DNA by wave scan and FTIR analysis. The amount of ethyl gallate present in the *A. nilotica* (L.) leaf extract was calculated using HPLC and represented in gram equivalence of ethyl gallate. The acute toxicity profile of ethyl gallate in the *A. nilotica* (L.) leaf extract was analyzed in albino Wistar rats. Measurement of liver and kidney function markers, total proteins and glucose were determined in the serum. Statistical analysis was done using statistical package for social sciences (SPSS) tool version 16.0.

**Results:**

Ethyl gallate was found to be effective at 100 μg/mL concentration by inhibiting the free radical mediated damage to BSA and pBR322 DNA. We also found that the interaction of ethyl gallate and *A. nilotica* (L.) leaf extract to CT-DNA occurs through intercalation. One gram of *A. nilotica* (L.) leaf extract was found to be equivalent to 20 mg of ethyl gallate through HPLC analysis. Based on the acute toxicity results, *A. nilotica* (L.) leaf extract and ethyl gallate as well was found to be non-toxic and safe.

**Conclusions:**

Results revealed no mortality or abnormal biochemical changes *in vivo* and the protective effect of *A. nilotica* (L.) leaf extract and ethyl gallate on DNA and protein against oxidative stress *in vitro*. Hence, *A. nilotica* (L.) leaf extract or ethyl gallate could be used as potential antioxidants with safe therapeutic application in cancer chemotherapy.

## Background

Oxidative stress due to reactive oxygen species/reactive nitrogen species (ROS/RNS) is associated with many diseases like cancer, cardiovascular disease, diabetes, arthritis and neurodegeneration [1]. ROS generation damages the biological macromolecules such as proteins, DNA, polysaccharides and lipids. These free radicals are scavenged by compounds that are capable of donating a hydrogen atom or by activating the antioxidant enzymes [2]. Many synthetic antioxidants were produced to protect the biological macromolecules against oxidative damage. However, there has been an increasing concern regarding the toxic effect of the available antioxidants. Therefore, in recent years, the interest in natural substances from medicinal plants has increased enormously due to the alleviation of diseases by the neutralization of bio-macromolecular oxidation [3].

*Acacia nilotica* (L.) belonging to the family Fabaceae and sub-family Mimosoideae is a medium sized tree with a variety of medicinal uses. The species is widely distributed in various tropical and sub-tropical countries around the world. The plant is used traditionally in several parts of Chhattisgarh state of India for treating cancers of the mouth, bones and skin, and in West Africa for tumors of ears, eyes, or testicles [4]. Twigs of the plant are used as tooth brushes in many parts of India and Africa [5,6]. Various scientific reports suggest that *A. nilotica* (L.) is rich in bioactive compounds and nutrients to treat many diseases such as cold, bronchitis, asthma, diabetes, diarrhoea, dysentery, blindness, bleeding piles and leucoderma to name a few [7,8]. *A. nilotica* (L.) has been reported to exhibit potent antioxidant activity as well, and it has been shown to be significant in comparison with quercetin, tocopherol, ascorbic acid and catechin. Leaves of *A. nilotica* (L.) are found to be rich in rutin and apigenin-6, 8-bi's-C-β-D-glucopyranoside [4,9]. Kalaivani *et al*. [10,11] has reported the antioxidant capacity of ethyl gallate isolated from the ethanol leaf extract of *Acacia nilotica* (L.) Wild. ex. Delile subsp. *indica* (Benth.) Brenan. Subsequently, the cytotoxic activity of ethyl gallate and *A. nilotica* (L.) leaf extract on HeLa cancer cells and Vero normal cells were established. Furthermore, ethyl gallate isolated from *Galla rhois* was reported to possess anticancer activity against human leukemia cell line through induction of apoptosis [12].

Ethyl gallate is a phenolic antioxidant compound present in foods with cancer prevention potential [13]. Therefore, the medicinal and protective properties of ethyl gallate isolated from *A. nilotica* (L.) leaf extract could be employed in the formulation of drugs. However, there is a paucity of literature information on the toxicity profile of *A. nilotica* (L.) leaf extract or ethyl gallate*.* The knowledge on their toxicity profile is essential in order to develop a potent drug from them. Thus, the objective was to determine the safety of *A. nilotica* (L.) leaf extract and ethyl gallate by assessing their dose-dependent toxicity profile on female albino Wistar rats. The attenuation of DNA or protein damage by Fenton’s system-generated free radicals was also evaluated. In addition, the nature of interaction of *A. nilotica* (L.) leaf extract and ethyl gallate on CT-DNA was examined by FTIR spectroscopy.

## Methods

### Reagents

The chemicals ethyl gallate, bovine serum albumin (BSA) and Calf thymus DNA (CT-DNA) were purchased from Sigma-Aldrich Chemical Co (St Louis, MO. USA). pBR322 DNA was purchased from GENEI (Bangalore, India). Biochemical kits were obtained from Span diagnostics (Surat, Gujarat, India), and all other reagents used were of analytical grade.

### Plant material and extraction

Leaves of *Acacia nilotica* (L.) Wild. ex. Delile subsp. *indica* (Benth.) Brenan were collected from Vellore district, Tamilnadu, India and identified by Dr. G.V.S. Murthy, Scientist-in-Charge, Botanical Survey of India, Southern Regional Centre, Tamilnadu Agricultural University, Coimbatore, India (Voucher number: 1035). *A. nilotica* (L.) dried leaves (100 g) were extracted exhaustively using ethanol by Soxhlet extraction yielding 40 g after evaporation as described in our previous report [4]. In addition, for validation of the presence of ethyl gallate in the leaves of *A. nilotica* (L.), 1 g of leaves were extracted by maceration with 20 mL of different solvents separately like methanol-acetonitrile-10 mM ammonium acetate containing 0.1% formic acid (10:25:65 v/v/v) according to the method of Gao *et al*. [14], ethanol and water. The supernatant was recovered after filtering the extract through Whatman No. 1 filter paper. The solvent present was evaporated in a vacuum rotary evaporator to obtain the dry extract. The dried extracts were dissolved in methanol-acetonitrile-10 mM ammonium acetate containing 0.1% formic acid (10:25:65 v/v/v) (1.0 mg/mL) and filtered through sterile 0.22 μm millipore filter and subjected to HPLC analysis.

### Protein damage assay

The protective effect of *A. nilotica* (L.) leaf extract and ethyl gallate were tested against Fenton’s system generated protein oxidation by the electrophoretic pattern on Sodium Dodecyl Sulphate-Polyacrylamide Gel Electrophoresis (SDS-PAGE) according to the method of Wang *et al*. [15] with slight modification. Reaction mixture containing 1 mg/mL of bovine serum albumin (BSA), Ferric chloride (FeCl_3_) (50 μM), hydrogen peroxide (H_2_O_2_) (1 mM) and ascorbic acid (100 μM) with or without *A. nilotica* (L.) leaf extract (100 μg/mL), ethyl gallate (100 μg/mL) or butylated hydroxyl toluene (BHT) (100 μg/mL) was made up to 1.2 mL in 20 mM potassium phosphate buffer of pH 7.4. The reaction mixture was incubated at 37°C for 3 h. Electrophoresis of the samples were carried out according to Laemmli’s method [16] in 12% SDS-PAGE and analyzed using the gel documentation system, AlphaImager HP, Cell Biosciences (Santa Clara, CA).

### DNA damage assay

The protective effect of *A. nilotica* (L.) leaf extract and ethyl gallate were tested for deoxyribonucleic acid (DNA) damage based on the method of Lee *et al*. [17]. The pBR322 plasmid DNA (200 ng) was oxidized using Fenton’s system (Fe^3+^/H_2_O_2_/ascorbic acid) in the presence or absence of *A. nilotica* (L.) leaf extract or ethyl gallate or quercetin at 100 μg/mL concentration for 30 min at 37°C. After incubation of these compounds with DNA, 5 μL of all the samples were loaded along with gel loading dye in 1% agarose gel for electrophoresis. The gel was scanned using the gel documentation system, AlphaImager HP, Cell Biosciences (Santa Clara, CA).

### DNA interaction by FTIR and UV analysis

FTIR spectroscopy is widely used in recent years for the interaction studies of DNA with natural compounds. A solution of CT-DNA was made with 10 mM Tris-HCl buffer of pH 7.4 and its purity was verified by its absorbance at 260 and 280 nm. Different concentrations of DNA (0.1 to 1 mg mL^-1^) were analyzed for its binding capacity with a single concentration of *A. nilotica* (L.) leaf extract or ethyl gallate [18].

IRAffinity-1 FTIR spectrophotometer (Shimadzu, Columbia, Maryland, USA) was used for recording the spectra using DTGS detector, Ni-Chrome source and KBr beam splitter. One hundred scans for each sample with 4 cm^-1^ resolution were recorded and evaluated using OMNIC software. The interaction of *A. nilotica* (L.) leaf extract and ethyl gallate with CT-DNA was evaluated by comparing the shift in the spectrum formed individually or as complexes. A UV-Visible spectrum was also recorded by wave scan range from 200 to 800 nm using Systronics AU-2701 UV-Vis double beam spectrophotometer (Gujarat, India).

### HPLC analysis

A stock of 1 mg/mL of the *A. nilotica* (L.) leaf extract was prepared using 0.1% ethanol. From this, 100 μL was taken and diluted to 3 mL with methanol. Wave scan analysis was carried out using Systronics AU-2701 UV-Vis double beam spectrophotometer in the wave length ranging from 200 to 800 nm. The peak obtained was compared with that of ethyl gallate. Following this, *A. nilotica* (L.) leaf extract was dissolved in HPLC grade methanol at a concentration of 1.0 mg/mL, filtered through 0.22 μm filter and subjected to HPLC Schimadzu isocratic system equipped with Luna C18 column. Separation was achieved using acetonitrile/water and the peak obtained was compared with the pure ethyl gallate as standard at 272 nm.

Validation of the presence of ethyl gallate in *A. nilotica* (L.) leaf extract with different solvents were also done using YOUNGLIN HPLC instrument Acme 9000 with vacuum degasser and mixer. The instrument was equipped with a gradient pump SP930D, a UV/Vis detector UV730D and a Kromasil 100-5C18 column with a length of 250 x 4.6 mm. Data was integrated by the software YOUNGLIN Autochro-3000 chromatograph data system. Separation was achieved by isocratic mobile phase consisting of methanol-acetonitrile-10 mM ammonium acetate containing 0.1% formic acid (10:25:65, v/v/v) with a flow rate of 0.5 mL/min. Peak area of the sample was compared with that of the standard at 291 nm.

### Animals

Forty eight female albino Wistar rats of six to eight weeks old were obtained from the Institutional animal house, VIT University, Vellore, Tamilnadu, India. All animals were maintained under standard conditions of temperature (28 ± 2°C) and light (12 h light/dark cycles). The animals were housed in polypropylene cages (45 × 24 × 15 cm) and fed with standard diet pellets and water *ad libitum*. Animals were handled according to the University and Institutional Regulations, administered by the Animal Ethical Committee, VIT University. The protocols performed on the animals were approved and conducted in accordance with the National Institute of Health Guide (VIT/IAEC/V/017/2012).

### Acute toxicity study

Rats were divided into eight groups based on their body weights. Animals were deprived of food but not water, 15 h prior to the administration of test substances. Toxicity was assessed by oral administration of 1 mL of *A. nilotica* (L.) leaf extract and ethyl gallate by gavage feeding and monitored for any mortality up to 14 days. Group 1 rats served as control receiving 1.0 mL of the vehicle (0.1% ethanol); Group 2 rats received *A. nilotica* (L.) leaf extract (250 mg/kg body weight, equivalent to 5 mg/kg ethyl gallate); Group 3 rats received *A. nilotica* (L.) leaf extract (500 mg/kg body weight, equivalent to 10 mg/kg ethyl gallate); Group 4 rats received *A. nilotica* (L.) leaf extract (1000 mg/kg body weight, equivalent to 20 mg/kg ethyl gallate); Group 5 rats received *A. nilotica* (L.) leaf extract (2000 mg/kg body weight); Group 6 rats received ethyl gallate (5 mg/kg body weight); Group 7 rats received ethyl gallate (10 mg/kg body weight); Group 8 rats received ethyl gallate (20 mg/kg body weight).

Body weights were recorded on 0^th^ and 14^th^ day for each group and all rats were decapitated after an overnight fast [19]. Liver and kidney tissues were removed and rinsed with saline solution, observed for any lesions and weighed. Blood was collected for the assessment of total serum proteins, glucose, liver and kidney function markers such as aspartate aminotransferase (AST), alanine aminotransferase (ALT) and alkaline phosphatase (ALP) activities; and total bilirubin and creatinine levels in the serum using Span diagnostic reagent kits [20]. Serum was separated from the collected blood after centrifugation at 3000 rpm for 10 min without any anticoagulant.

### Statistical analysis

All quantitative measurements were expressed as mean ± standard error (SEM). Statistical analysis was performed by one way analysis of variance (ANOVA) followed by Duncan’s Multiple Range Test (DMRT) using Statistical Package for Social Sciences (SPSS) Version 16.0. *p* < 0.05 were considered statistically significant.

## Results and discussion

Although medicinal plants and the plant derived bioactive compounds appear to contribute to the prevention and progression of diseases, they are not nutrients. Hence, the knowledge of their safety and side-effects, if any, are important considerations for their effective use in disease management. In this regard, the general toxicity of medicinal plants and their derivatives needs to be validated from a toxicological point of view. In our previous report, we have determined the titres of phenolics in the *A. nilotica* (L.) leaf extract and assessed their antioxidant activity [4]. Ethyl gallate, a major phenolic compound obtained from *A. nilotica* (L.) leaf extract has also been shown to inhibit the growth of cancer cells *in vitro*. In this report, *A. nilotica* (L.) leaf extract and ethyl gallate was evaluated for their protection against Fenton’s system generated free radical damage on biological macromolecules like DNA and protein. The nature of interaction of *A. nilotica* (L.) leaf extract and ethyl gallate to CT-DNA was analyzed using FTIR and UV spectroscopy. The toxicological profile was also studied using albino Wistar rats.

### Protection of BSA and DNA damage against oxidative stress

**Figure 1:**
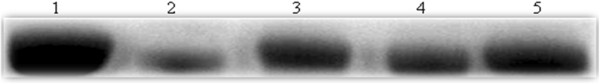
**Antioxidant activity of *A. nilotica *(L.) leaf extract and ethyl gallate against protein damage.** Lane 1 - BSA, 2 - BSA + Fenton, 3 - BSA + Fenton + extract (100 μg/mL), 4 - BSA + Fenton + ethyl gallate (100 μg/mL), 5 - BSA + Fenton + BHT (100 μg/mL).

**Figure 2:**
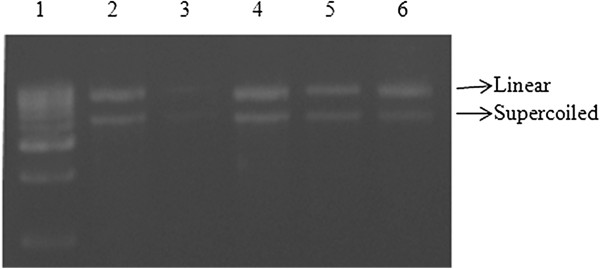
**Antioxidant activity of *A. nilotica *(L.) leaf extract and ethyl gallate against DNA damage.** Lane 1 - DNA ladder, 2 - pBR322 DNA, 3 - DNA + Fenton, 4 - DNA + Fenton + extract (100 μg/mL), 5 - DNA + Fenton + ethyl gallate (100 μg/mL) and 6 - DNA + Fenton + quercetin (100 μg/mL).

The protective effect of ethyl gallate was compared with *A. nilotica* (L.) leaf extract to assess their protection on oxidation of BSA and pBR322 DNA against the generation of hydroxyl radicals through Fenton’s system. As shown in Figure 1, the protein (BSA) appeared to have been degraded by the deleterious effect of free radicals generated by Fenton’s system. Inhibitory effect of *A. nilotica* (L.) leaf extract and ethyl gallate on protein oxidation could be recognized when the intensity of the protein band was retained on 12% SDS-PAGE; the effects were comparable with that of the standard BHT. Moreover, a prominent damage was also observed in DNA treated with Fenton’s system (Figure 2). *A. nilotica* (L.) leaf extract and ethyl gallate as well attenuated the DNA damage caused by the Fenton’s system. The effective antioxidant activity of the test substances was evident from the integrity of the supercoiled DNA when compared with that of the standard quercetin.

Hydroxyl radicals are well known cause for damaging biological macromolecules leading to mutation, cancer and age-related diseases [15]. Polyphenols are known to inhibit the adverse effects of oxidative stress through their anticancer and antimutagenic properties [21]. The results of this study show that *A. nilotica* (L.) leaf extract and ethyl gallate act as active scavengers of hydroxyl radicals, thereby protecting BSA and pBR322 DNA, without causing any toxicity. These results are in agreement with the previous work of Singh *et al*. [22] using *A. nilotica* (L.) green pod extracts against DNA damage.

### FTIR and wave scan analysis

**Figure 3:**
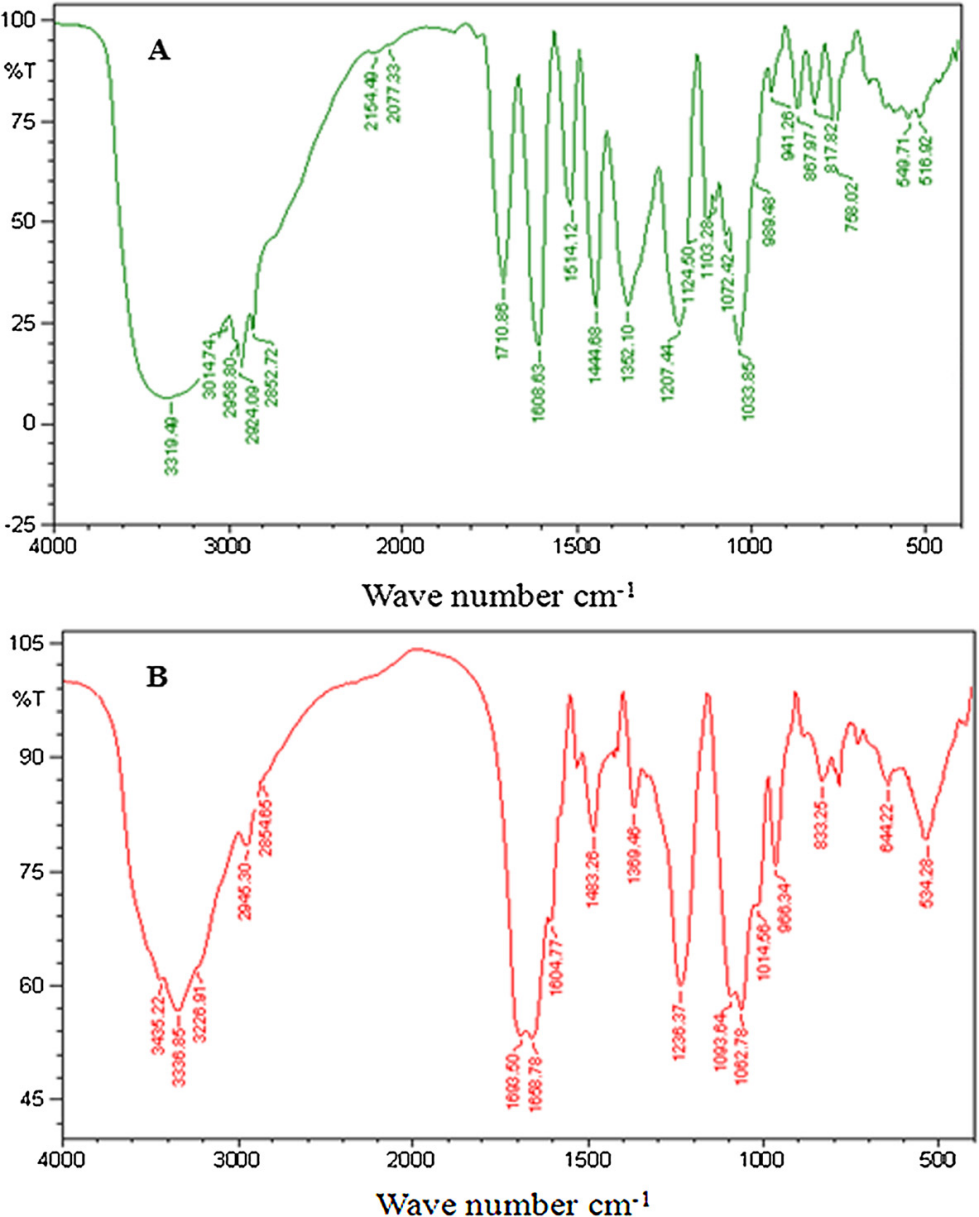
**FTIR spectra (A) *A. nilotica *** **(L.) leaf extract (B) *A. nilotica *** **(L.) leaf extract with varying concentrations of DNA.**

**Figure 4:**
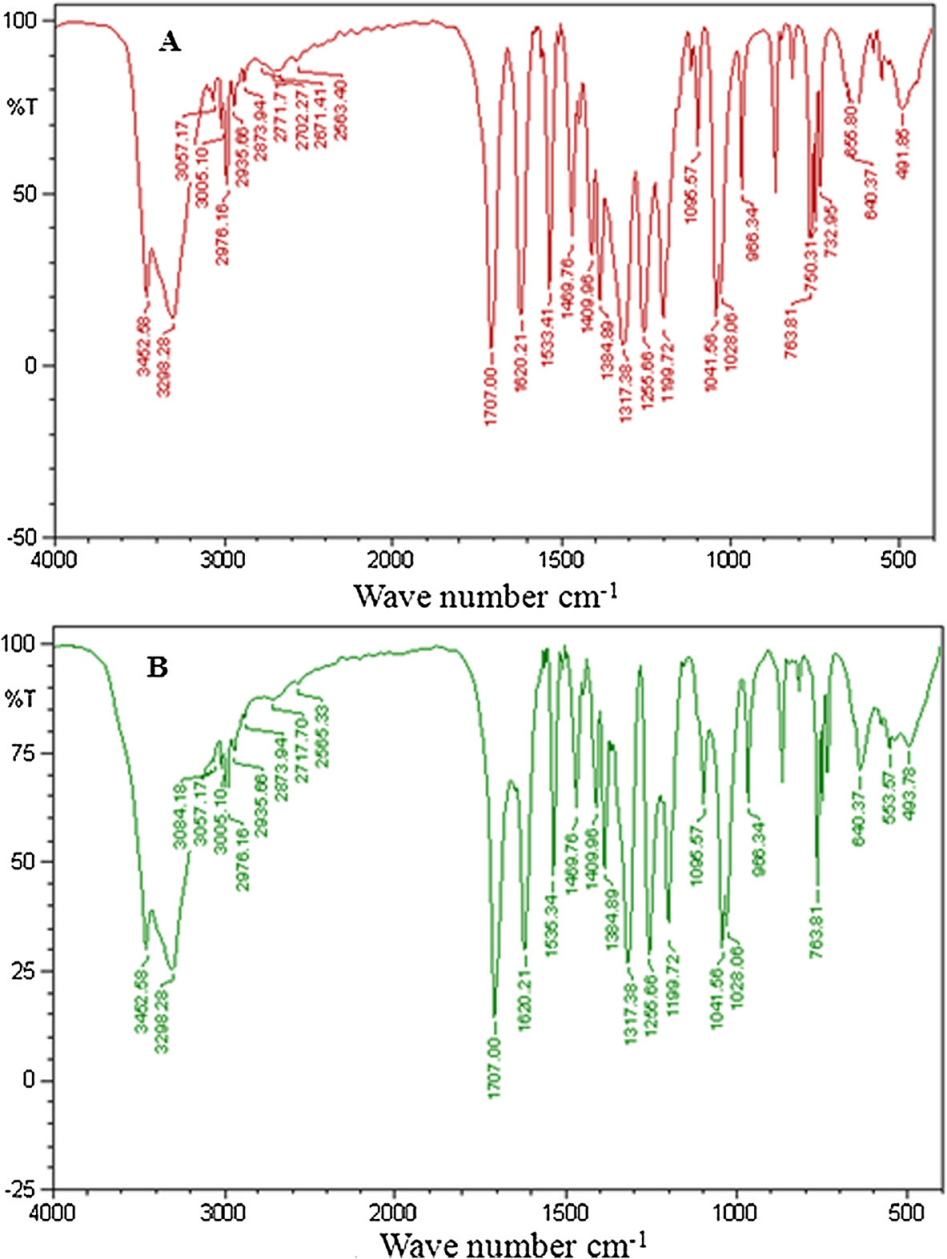
FTIR spectra (A) ethyl gallate (B) ethyl gallate with varying concentrations of DNA.

FTIR spectroscopy is one of the widely used tools for the characterization of drug-biomolecule interaction. Teel *et al.*[23] has reported that plants exhibit antimutagenic/anticarcinogenic activity based on the interaction of active constituents present in the plant with DNA by blocking the sites of DNA to damage caused by reactive mutagenic moieties. Based on this aspect, Boubaker *et al.*[24] has reported the antimutagenic effect of *A. salicina* leaf extract through complex formation between the mutagens, 2-aminoanthracene (2-AA) and benzo[a]pyrene [B(a)P]. He also found that the extract was more potent in inhibiting the frame-shift mutation. Based on the protective effect of *A. nilotica* (L.) leaf extract and ethyl gallate on pBR322 DNA against Fenton’s system generated oxidative stress, the authors continued to study the interaction of *A. nilotica* (L.) leaf extract and ethyl gallate to CT-DNA. FTIR spectra of CT-DNA and its complexes with *A. nilotica* (L.) leaf extract and ethyl gallate are shown in Figures 3 and 4. Absorption at 1710 cm^-1^ is attributable to guanine, 1663 cm^-1^ to thymine, 1608 cm^-1^ to adenine and 1491 cm^-1^ to cytosine [25]. *A. nilotica* (L.) leaf extract and CT-DNA formed a complex, as evidenced by an increase in the intensity of vibration with characteristic positive peaks at 1693 cm^-1^ for guanine, 1658 cm^-1^ for thymine, 1604 cm^-1^ for adenine and 1483 cm^-1^ for cytosine (Figure 3A and B). In addition, a phosphate asymmetric stretch was noticed as the absorption shifted from 1225 cm^-1^ to 1236 cm^-1^. Furthermore, a symmetric stretch was found as the peak shifted from 1088 cm^-1^ to 1093 cm^-^1. Similar patterns of binding were observed by Tyagi *et al*. [26] for vincristine and DNA complex in FTIR spectra. As shown in Figure 4A and B, upon addition of CT-DNA to ethyl gallate, we observed a variation in the intensity of 1469 cm^-1^ indicating the positive peak vibration of cytosine binding to ethyl gallate [25]. In addition, a moderate shift and the intensity vibration of 1533 cm^-1^ to 1535 cm^-1^ indicates the structural change that occurred in CT-DNA. Moreover, the appearance (553 cm^-1^) or disappearance (750 cm^-1^, 732 cm^-1^ and 655 cm^-1^) of peaks demonstrates a plausible interaction of ethyl gallate to CT-DNA [18].

**Figure 5:**
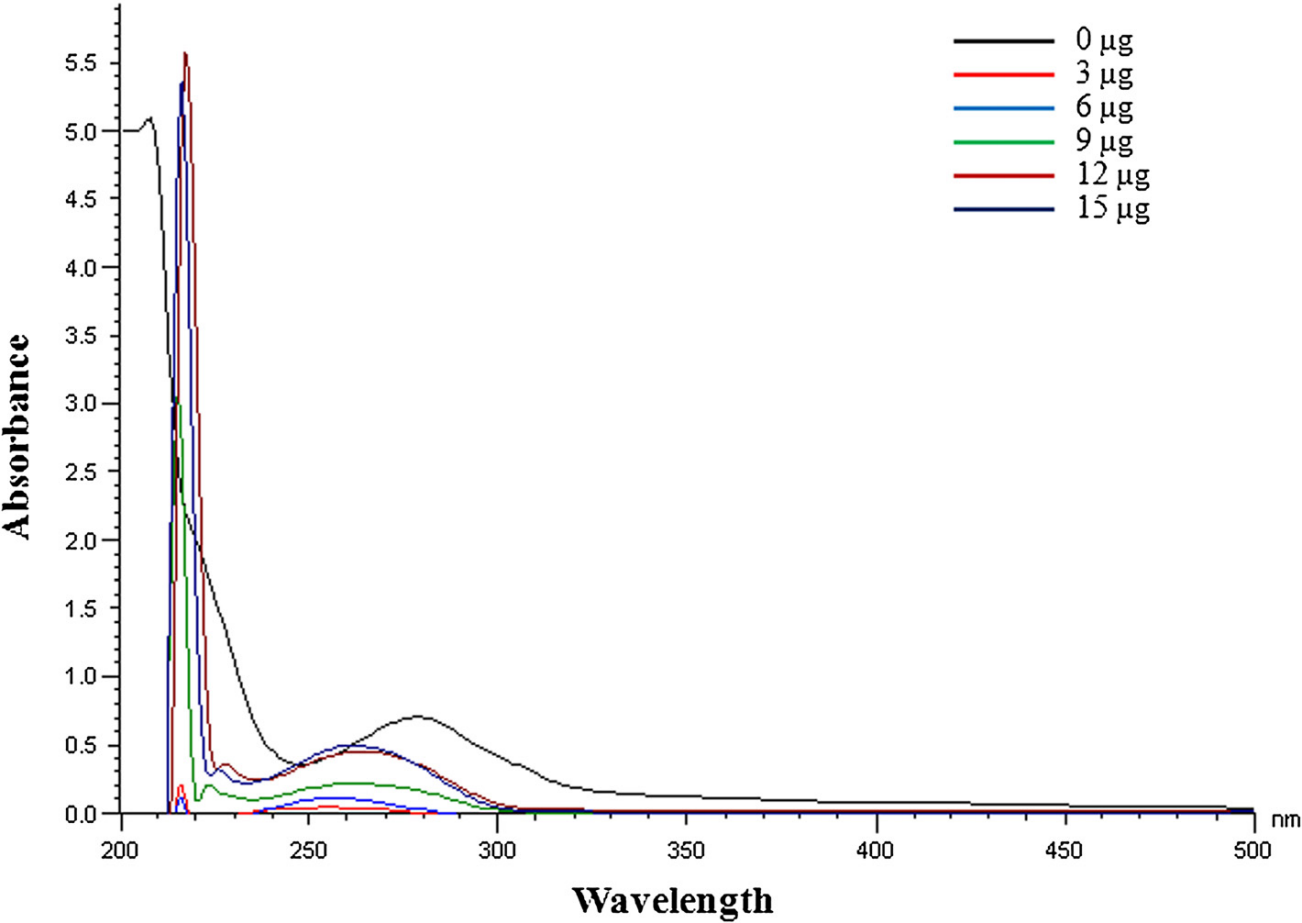
**UV-Visible spectrum of *A. nilotica *** **(L.) leaf extract and DNA complex.**

**Figure 6:**
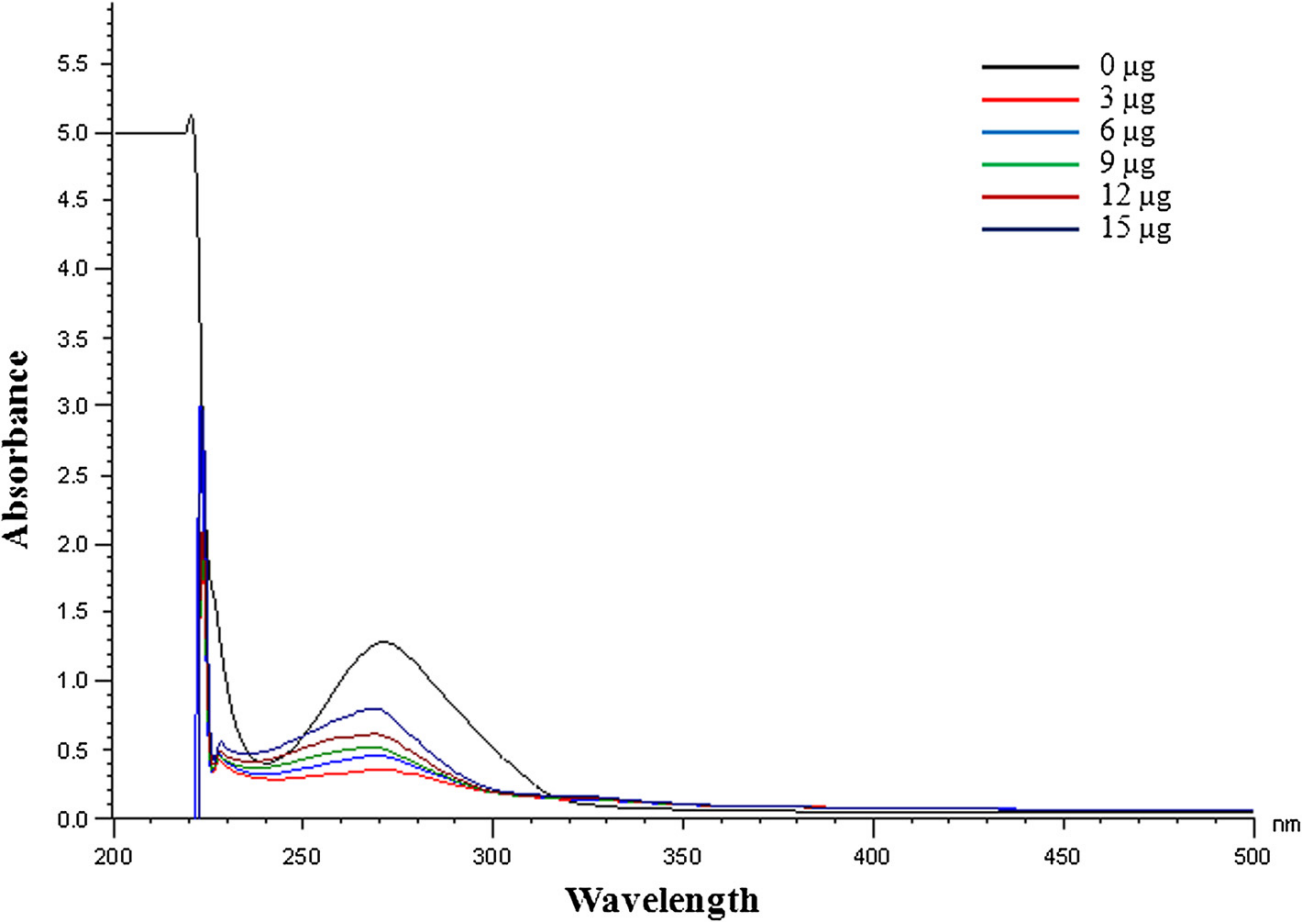
UV-Visible spectrum of ethyl gallate and DNA complex.

UV-Vis spectral analysis of *A. nilotica* (L.) leaf extract and ethyl gallate to varying concentrations of CT-DNA are shown in Figures 5 and 6. The maximum absorbance for *A. nilotica* (L.) leaf extract was reduced with a shift from 278 nm to 260 nm indicating hypochromism with hypsochromic or blue shift effect (Figure 5) [27]. Similarly, the maximum absorbance for ethyl gallate becomes lesser with a shift from 271 nm to 268 nm indicating hypochromism with blue shift effect (Figure 6). This shift in wavelength indicates that the interaction of *A. nilotica* (L.) leaf extract and ethyl gallate to DNA is by intercalation. The results also suggest that if the concentration of DNA is further increased, it may lead to helical destabilization [28]. These observations suggest that both *A. nilotica* (L.) leaf extract and ethyl gallate could offer protection to pBR322 DNA from oxidization or damage by forming a complex with DNA as well as by free radical scavenging mechanism.

### HPLC analysis

**Figure 7:**
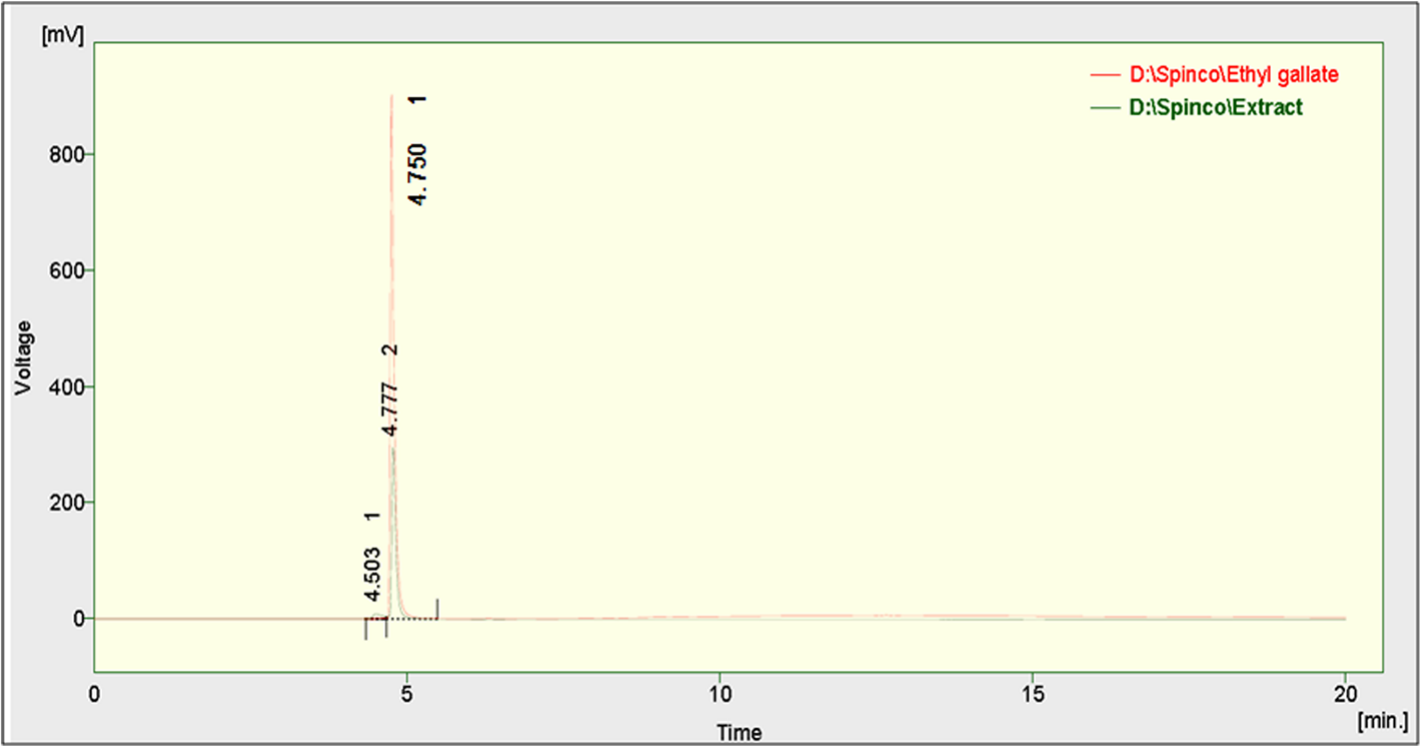
**HPLC chromatograms of ethyl gallate and *A. nilotica *(L.) leaf extract.***A. nilotica* (L.) leaf extract by Soxhlet extraction using ethanol as solvent at 272 nm.

**Figure 8:**
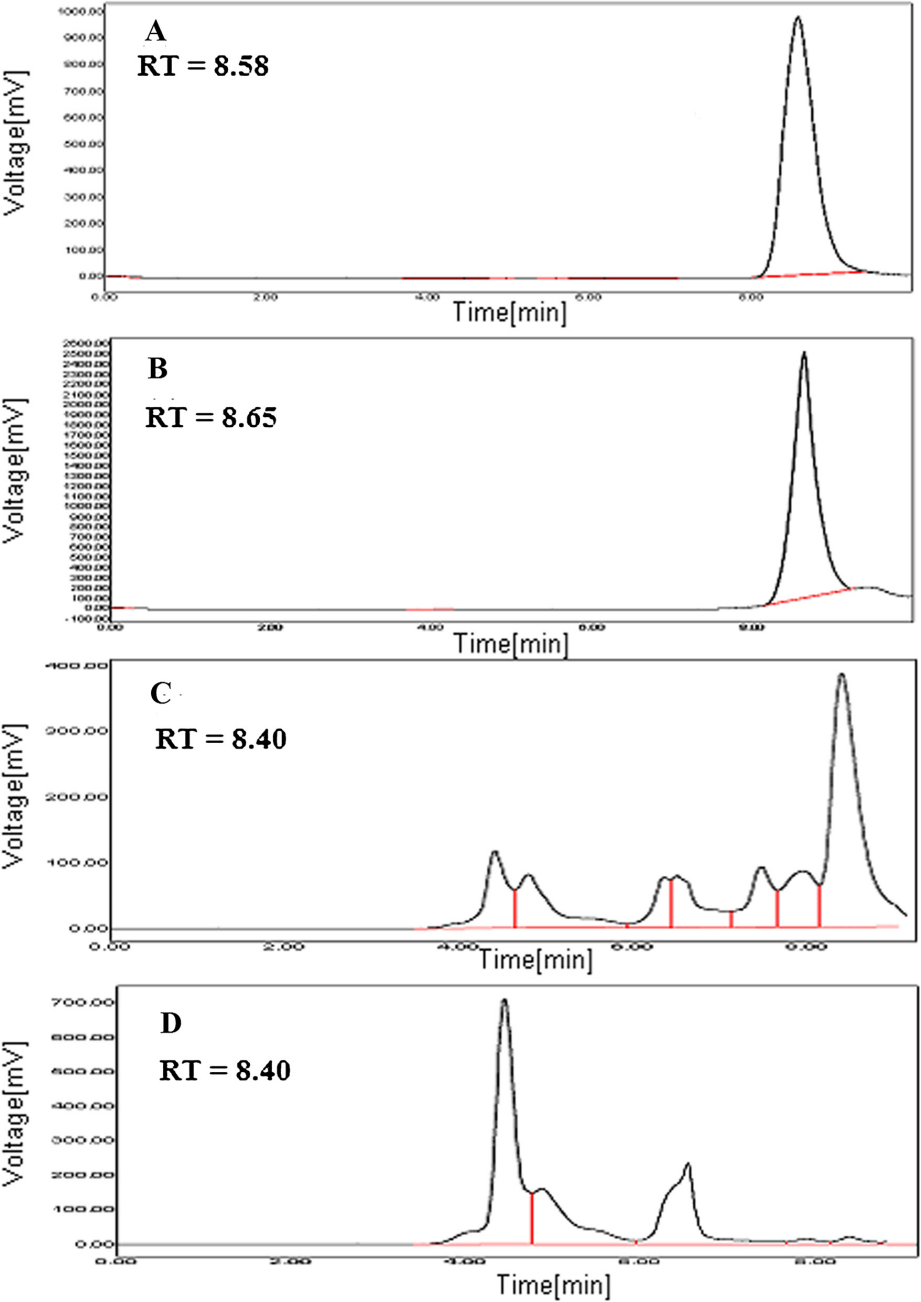
**HPLC chromatograms of ethyl gallate and *A. nilotica *(L.) leaf extract. (A)** ethyl gallate, representative chromatograms of *A. nilotica* (L.) leaf extract by maceration using **(B)** methanol-acetonitrile-10 mM ammonium acetate containing 0.1% formic acid (10:25:65 v/v/v) **(C)** ethanol and **(D)** aqueous solvents at 291 nm.

In this study, the amount of ethyl gallate present in the *A. nilotica* (L.) leaf extract was calculated based on their retention time in HPLC analysis (Figure 7). 1 g of *A. nilotica* (L.) leaf extract was equivalent to 20 mg of ethyl gallate when ethanol was used as a solvent by Soxhlet extraction. Similarly, when the leaves were macerated with methanol-acetonitrile-10 mM ammonium acetate containing 0.1% formic acid (10:25:65, v/v/v), ethanol and water yielded around 18.05 mg/g dry extract, 3.28 mg/g dry extract and 0.14 mg/g dry extract of ethyl gallate respectively. The present result thus authenticates the presence of ethyl gallate in *A. nilotica* (L.) leaf extract by maceration as well using different solvents (Figure 8).

### Acute toxicity study in rats

An acute toxicity study was performed to evaluate the safe administrable doses of *A. nilotica* (L.) leaf extract or ethyl gallate in female albino Wistar rats for 14 days. Generally, any test substance showing an LD_50_ of 1000 mg/kg through oral mode can be considered safe [29]. Group 1 received the vehicle alone (0.1% ethanol); groups 2 to 4 received *A. nilotica* (L.) leaf extract of 250, 500, 1000 mg/kg body weight (ethyl gallate equivalent of 5, 10 and 20 mg/kg, respectively); group 5 received 2000 mg/kg body weight of *A. nilotica* (L.) leaf extract or ethyl gallate equivalent of 40 mg/kg; and groups 6 to 8 received ethyl gallate alone at doses of 5, 10 and 20 mg/kg body weight respectively. Administration of these test substances orally did not show any mortality in the treated groups. Hence, the LD_50_ could not be determined for the administered doses. Previous report shows that *A. nilotica* (L.) flower extract was found to be non-toxic up to a dose of 2000 mg/kg [30]. Similarly, ethyl acetate fruit extract of *A. nilotica* (L.) upon oral administration to mice showed LD_50_ value of 7393.4 mg/kg [31]. In contrast, 20-100% mortality was reported in rats treated with 50-500 mg/kg *A. nilotica* (L.) fruit extract on intraperitoneal administration [32]. However, in the present investigation no mortality was observed for *A. nilotica* (L.) leaf extract on oral administration to rats.

Changes in body weight or organ weight may also suggest adverse side effects of test substances. Table 1 shows the changes in body weight for all the experimental groups. A significant increase in body weight was observed in all the treatment groups when compared with the control group. The loss of body weight can, often, be a sensitive indicator of toxicity after exposure to toxic substances [33,34]. Although a decrease in body weight was reported in rats fed with 2% and 8% *Acacia* in the diet for 2 and 4 weeks, the condition was reversed in a few days after termination of the treatment without any mortality [35]. In the present study, maximum gain in body weight was observed in group 3 administered with a dose of 500 mg/kg body weight of *A. nilotica* (L.) leaf extract (ethyl gallate equivalent of 10 mg/kg) and in group 6 administered with 5 mg/kg body weight of ethyl gallate. Similarly, the liver weight was found to be higher in group 5 administered with 2000 mg/kg body weight of *A. nilotica* (L.) leaf extract and in group 6 administered with 5 mg/kg body weight of ethyl gallate. Nonetheless, these changes did not lead to any mortality of the animals.

**Table 1:** **Effect of *A. nilotica *** **(L.) leaf extract and ethyl gallate on weight analysis in rats**

**Group**	**Body weight gain (g)**	**Liver weight (g)**	**Kidney weight (g)**
**1**	10.00 ± 1.0^a^	5.44 ± 0.16^a,b^	1.28 ± 0.06^a^
**2**	15.33 ± 1.52^c,d^	5.29 ± 0.39^a^	1.27 ± 0.18^a^
**3**	21.66 ± 1.15^e^	6.76 ± 0.43^b,c^	1.49 ± 0.12^a,b^
**4**	12.00 ± 1.73^a,b^	7.01 ± 0.95^c^	1.63 ± 0.06^b^
**5**	17.66 ± 2.30^c,d^	8.82 ± 0.97^d^	2.85 ± 0.15^c^
**6**	18.66 ± 1.15^c^	7.49 ± 0.86^c^	2.66 ± 0.16^c^
**7**	12.66 ± 1.55^b^	7.11 ± 0.97^c^	1.47 ± 0.09^a,b^
**8**	14.33 ± 1.15^b,c^	6.75 ± 0.81^b,c^	1.42 ± 0.19^a,b^

Each value represents mean ± SEM in each group. Values not sharing a common superscript (a-e) differ significantly with each other (*p* < 0.05, Duncan’s multiple range test (DMRT)). Group 1 - Control, Groups 2-5 (*A. nilotica* (L.) leaf extract) - 250, 500, 1000 and 2000 mg/kg body weight, Groups 6-8 (ethyl gallate) - 5, 10 and 20 mg/kg body weight.

### Biochemical analysis

**Figure 9:**
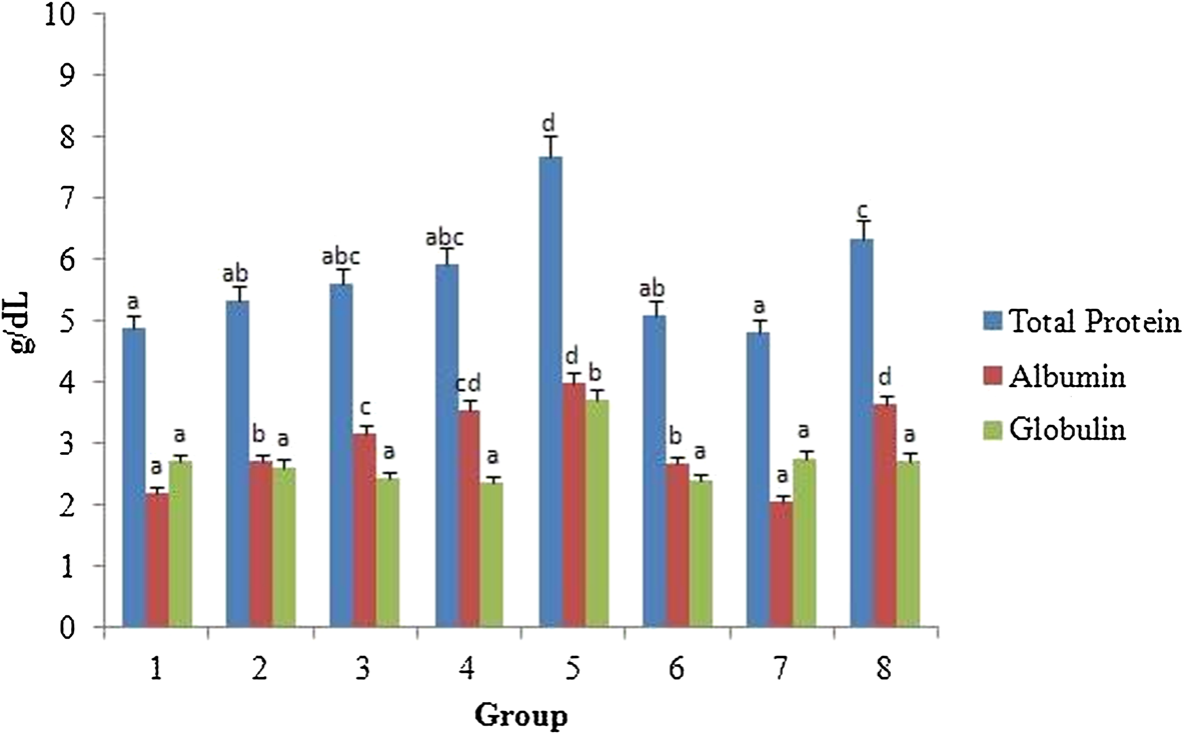
**Effect of *A. nilotica *(L.) leaf extract and ethyl gallate on total serum protein in rats.** Each value represents mean ± SEM in each group. Values not sharing a common superscript (a-d) differ significantly with each other (*p* < 0.05, Duncan’s multiple range test (DMRT)). Group 1 - Control, Groups 2-5 (*A. nilotica* (L.) leaf extract) - 250, 500, 1000 and 2000 mg/kg body weight, Groups 6-8 (ethyl gallate) - 5, 10 and 20 mg/kg body weight.

Biochemical changes in the blood are important indicators of the toxicity profile of any pharmaceutically important compounds. An increase in the level of total serum protein or glucose is an indicator of kidney impairment [36]. As depicted in Figure 9, the levels of total serum proteins such as total protein, albumin and globulin were found to remain within the normal ranges without any clinical significance [37]. The level of total serum proteins was found to be significantly higher in all the treatment groups when compared with the control (*p* < 0.05). The increase in total serum proteins was directly proportional to the increase in the concentration of *A. nilotica* (L.) leaf extract or ethyl gallate. The maximum protein level was found in rats treated with the *A. nilotica* (L.) leaf extract at 2000 mg/kg body weight (group 5) and ethyl gallate at 20 mg/kg body weight (group 8).

**Figure 10:**
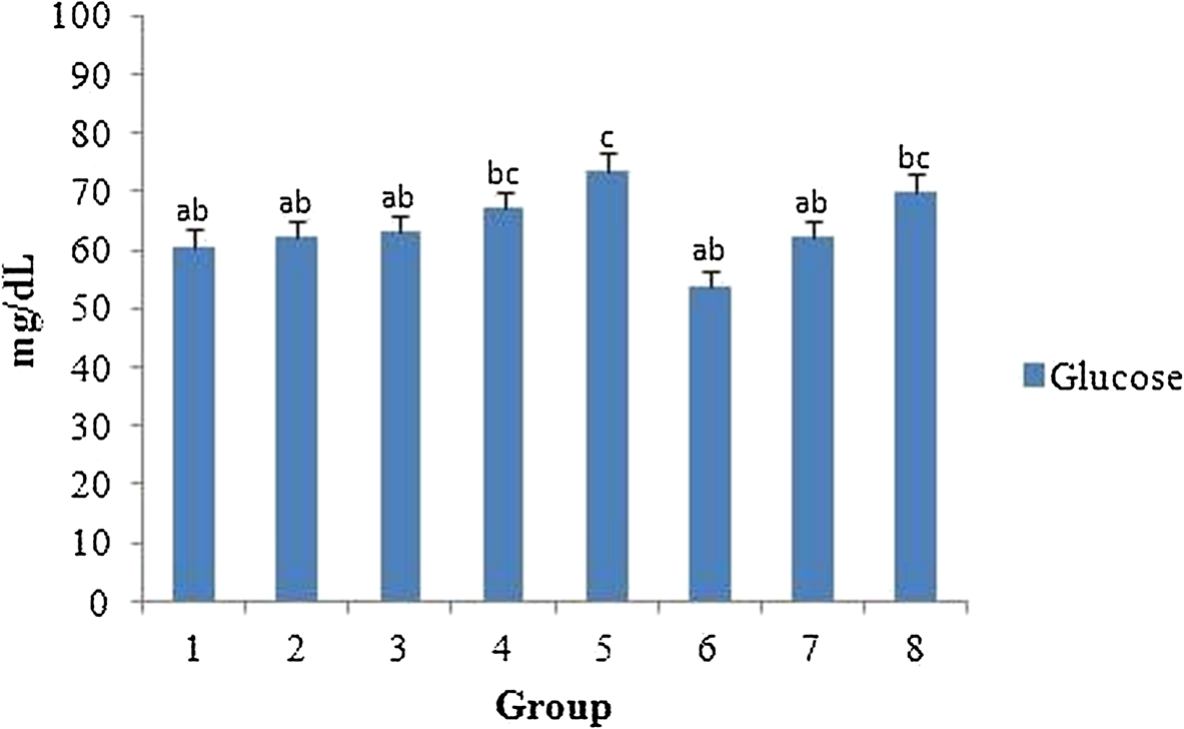
**Effect of *A. nilotica *(L.) leaf extract and ethyl gallate on serum glucose level in rats.** Each value represents mean ± SEM in each group. Values not sharing a common superscript (a-c) differ significantly with each other (*p* < 0.05, Duncan’s multiple range test (DMRT)). Group 1 - Control, Groups 2-5 (*A. nilotica* (L.) leaf extract) - 250, 500, 1000 and 2000 mg/kg body weight, Groups 6-8 (ethyl gallate) - 5, 10 and 20 mg/kg body weight.

Figure 10 shows the serum glucose level in all the treated groups. No significant difference in glucose levels was observed in all the treated groups when compared with the control, except at the highest concentration of *A. nilotica* (L.) leaf extract (2000 mg/kg body weight) or ethyl gallate (20 mg/kg body weight) tested. Nevertheless, the results obtained did not suggest any toxicity since all the values fall within the ranges of normalcy. The range of normalcy for total protein, albumin and glucose are reported to be 5.6-7.6 g/dL, 3.4-4.8 g/dL and 50-135 mg/dL respectively [38]. One report, however, on the methanolic leaf extract of *A. nilotica* (L.) was found to be hypoglycaemic on streptozotocin induced diabetic rats [39].

**Figure 11:**
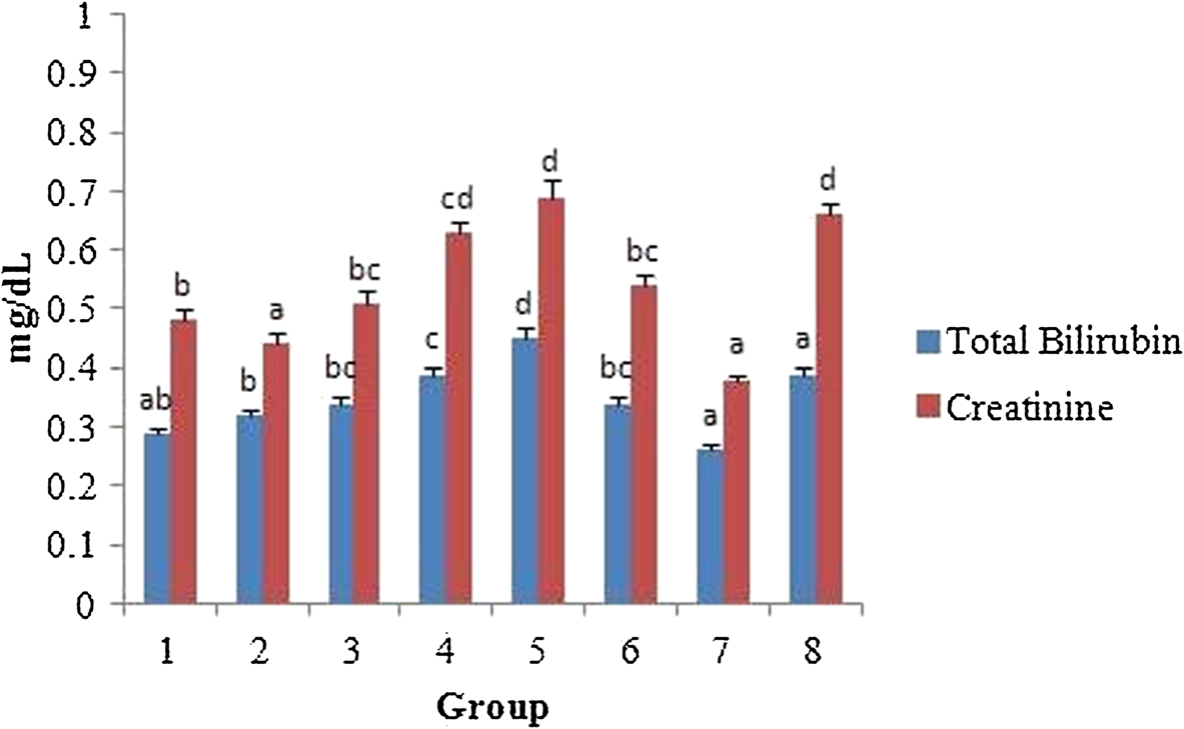
**Effect of *A. nilotica *(L.) leaf extract and ethyl gallate on bilirubin and creatinine in rats.** Each value represents mean ± SEM in each group. Values not sharing a common superscript (a-d) differ significantly with each other (*p* < 0.05, Duncan’s multiple range test (DMRT)). Group 1 - Control, Groups 2-5 (*A. nilotica* (L.) leaf extract) - 250, 500, 1000 and 2000 mg/kg body weight, Groups 6-8 (ethyl gallate) - 5, 10 and 20 mg/kg body weight.

**Figure 12:**
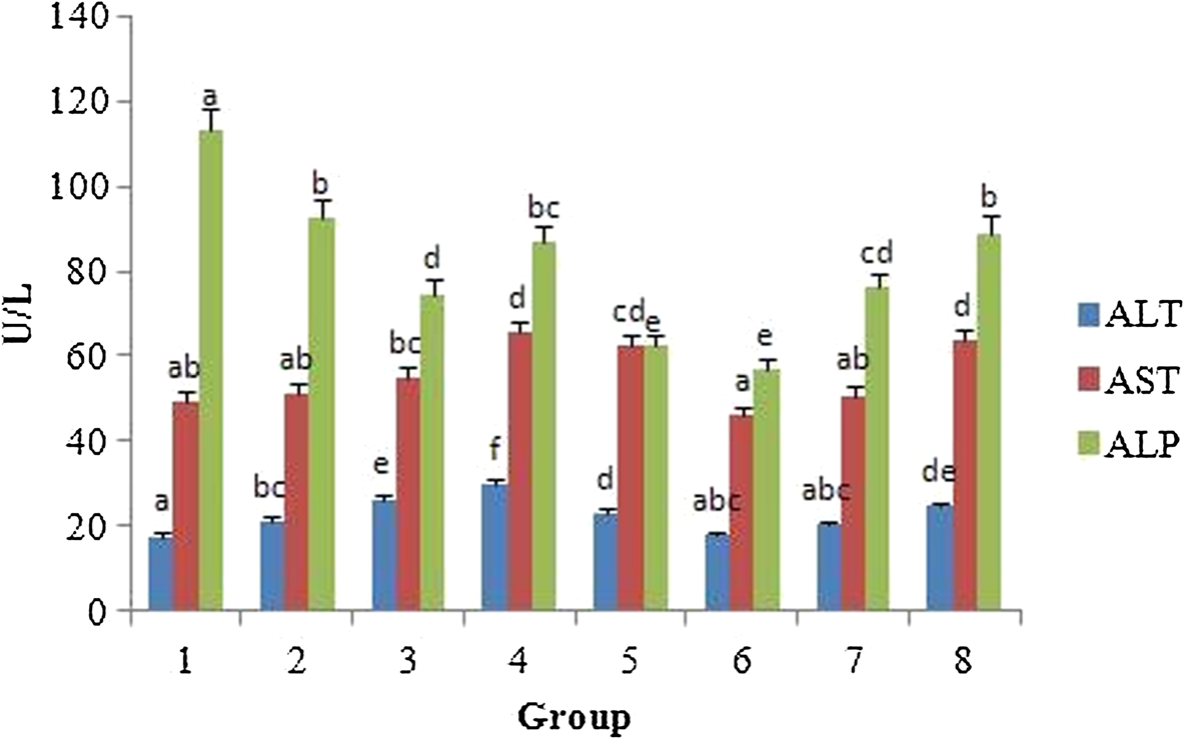
**Effect of *A. nilotica *(L.) leaf extract and ethyl gallate on serum liver markers in rats.** Each value represents mean ± SEM in each group. Values not sharing a common superscript (a-f) differ significantly with each other (*p* < 0.05, Duncan’s multiple range test (DMRT)). Group 1 - Control, Groups 2-5 (*A. nilotica* (L.) leaf extract) - 250, 500, 1000 and 2000 mg/kg body weight, Groups 6-8 (ethyl gallate) - 5, 10 and 20 mg/kg body weight.

Toxicity to the liver or kidney tissues has been reported for some phytotherapeutic products such as pyrrolizidine alkaloids, β-asarone, estragole etc. [40-43]. The markers such as AST, ALT and ALP were evaluated for liver function. The presence of these enzymes in the cytosol or mitochondria indicates serious hepatocellular damage or changes in the membrane permeability [44]. Figures 11 and 12 shows the levels of total bilirubin, creatinine and specific liver markers such as AST, ALT and ALP in the serum of control and experimental groups. The total serum bilirubin level was found to increase monotonically in proportion with the increase in concentration of the test substances in comparison with the control. Similarly, the creatinine level was also found to be elevated in the serum as the dose of the test substances increased. Creatinine is considered as a critical marker of damage for nephron function as it is eliminated by glomerular filtration.

No significant difference in the serum total protein, albumin, globulin and glucose was found between the rats fed with *A. nilotica* (L.) leaf extract on ethyl gallate equivalent basis and those fed with ethyl gallate alone. Significant differences in total bilirubin level, however, existed between the rats that received *A. nilotica* (L.) leaf extract, 500 mg/kg body weight (ethyl gallate equivalent of 10 mg/kg, 0.34 ± 0.01 mg/dL) and those receiving 10 mg/kg body weight of ethyl gallate (0.26 ± 0.01 mg/dL). The creatinine levels in the groups fed with *A. nilotica* (L.) leaf extract (group 2, 250 mg/kg body weight, 0.44 ± 0.02 mg/dL; and group 3, 500 mg/kg body weight, 0.51 ± 0.02 mg/dL) were significantly different (at 95% confidence) from ethyl gallate fed groups (group 6, 5 mg/kg body weight, 0.54 ± 0.02; and group 7, 10 mg/kg body weight, 0.38 ± 0.01 mg/dL).

No significant difference was found for AST between the groups receiving *A. nilotica* (L.) leaf extract (in ethyl gallate equivalence) and those receiving ethyl gallate. Likewise, no significant difference was found between any groups for ALP except for the groups treated with 250 mg/kg body weight (92.54 ± 4.27 U/L) of *A. nilotica* (L.) leaf extract (equivalent to 5 mg/kg ethyl gallate) and 5 mg/kg body weight (57 ± 2.63 U/L) of ethyl gallate at *p* < 0.05. However, significant difference was found for ALT between groups fed with 500 and 1000 mg/kg body weight of *A. nilotica* (L.) leaf extract (26.52 ± 1.23 and 30.05 ± 1.38 U/L) and 10 and 20 mg/kg of ethyl gallate (20.50 ± 0.94 and 24.67 ± 1.13 U/L).

Taken together, the liver and kidney function markers in the serum was found to be elevated with increase in the concentration of the test substances. When the liver marker values were compared on an equivalent basis of ethyl gallate, there were differences in liver markers between *A. nilotica* (L.) leaf extract and ethyl gallate, which could be attributed due to the presence of other constituents present in the *A. nilotica* (L.) leaf extract. Nevertheless, those changes do not appear to have any consequential clinical significance, since the biochemical indicators were found to be within the normal ranges [45]. Our results are also in line with the report by Kannan *et al.*[46] that the methanolic extract of the aerial part of *A. nilotica* (L.) offers protection against hepatotoxicity induced by acetaminophen in Wistar rats.

## Conclusions

*A. nilotica* (L.) leaf extract or ethyl gallate does not appear to possess any toxicity *in vivo* as evidenced by zero mortality, suggesting that a wide margin of safety is possible for the selected therapeutic doses. Secondly, biochemical changes in the serum did not show any signs of toxicity, indicating their safety. In addition, the absence of DNA or protein damage indicates that the test substances are effective antioxidants against the hydroxyl radicals generated by the Fenton’s system. FTIR and UV-Vis spectral results demonstrate, for the first time, that *A. nilotica* (L.) leaf extract and ethyl gallate has a binding affinity to DNA by intercalation. The mode of interaction of these substances to CT-DNA and the protection offered to pBR322 DNA and BSA suggests their potential for their use in cancer chemotherapy. Further work would involve the evaluation of the absorption and bioavailability of ethyl gallate and other constituents in the *A. nilotica* (L.) leaf extract through oral ingestion in rats.

## Abbreviations

FTIR: Fourier transform infrared; HPLC: High performance liquid chromatography; ROS: Reactive oxygen species; RNS: Reactive nitrogen species; CT-DNA: Calf-thymus deoxyribonucleic acid; SDS-PAGE: Sodium Dodecyl Sulphate-Polyacrylamide Gel Electrophoresis; BHT: Butylated hydroxyl toluene; FeCl_3_: Ferric chloride; H_2_O_2_: Hydrogen peroxide; BSA: Bovine serum albumin; LD_50_: Lethal dose 50; AST: Aspartate aminotransferase; ALT: Alanine aminotransferase; ALP: Alkaline phosphatise; RPM: Revolutions per minute; SEM: Standard error; ANOVA: Analysis of variance; DMRT: Duncan’s multiple range test; SPSS: Statistical package for social sciences.

## Competing interests

The authors declare that there are no competing interests.

## Authors’ contributions

RC conceived and designed the experiments. SM performed the experiments and wrote the manuscript. KT monitored the experiments and discussed the results. JA analyzed the data and reviewed the manuscript. All authors have read and approved the manuscript.

## Pre-publication history

The pre-publication history for this paper can be accessed here:

http://www.biomedcentral.com/1472-6882/14/257/prepub

## References

[B1] Donne DI Rossi R Colombo R Giustarini D Milzani A Biomarkers of oxidative damage in human disease Clin Chem 2006 52 601 623 16484333 10.1373/clinchem.2005.061408

[B2] Oboh G Antioxidant properties of some commonly consumed and underutilized tropical legumes Eur Food Res Tech 2006 224 61 65

[B3] Silva BM Andrade PB Valentaoo P Ferreres F Seabra RM Ferreira MA Quince (*Cydonia oblonga* Miller) fruit (pulp, peel and seed) and jam: antioxidant activity J Agric Food Chem 2004 52 4705 4712 15264903 10.1021/jf040057v

[B4] Kalaivani T Mathew L Free radical scavenging activity from leaves of *Acacia nilotica* (L.) Wild. ex Delile, an Indian medicinal tree Food Chem Toxicol 2010 48 298 305 19837122 10.1016/j.fct.2009.10.013

[B5] Hooda A Rathee M Singh J Chewing sticks in the era of toothbrush: a review Internet J Fam Pract 2009 9 1

[B6] Jansen PCM Cardon D Dyes and tannins Plant Resources of Tropical Africa 3. PROTA Foundation 2005 Wageningen, Netherlands: Backhuys Publishers 19 25

[B7] Ambasta SP The Wealth of India A dictionary of Indian raw materials and industrial products 1986 13 New Delhi: The useful plants of India, CSIR 198

[B8] Ali A Akhtar N Khan BA Khan MA Rasul A Zaman SU Khalid N Waseem K Mahmood T Ali L *Acacia nilotica*: a plant of multipurpose medicinal uses J Med Plants Res 2012 6 1492 1496

[B9] Seigler DS Phytochemistry of *Acacia-sensu lato* Biochem Syst Ecol 2003 31 845 873

[B10] Kalaivani T Rajasekaran C Mathew L Free radical scavenging, cytotoxic, and hemolytic activities of an active antioxidant compound ethyl gallate from leaves of Acacia nilotica (L.) wild. Ex. Delile subsp. Indica (Benth.) Brenan J Food Sci 2011 76 144 149 10.1111/j.1750-3841.2011.02243.x 22417526

[B11] Kalaivani T Rajasekaran C Suthindhiran K Mathew L Free radical scavenging, cytotoxic and haemolytic activities from leaves of *Acacia nilotica* (L.) Wild. ex. Delile subsp. indica (Benth.) Brenan Evid Based Complement Alternat Med 2010 2010 1 8 10.1093/ecam/neq060 PMC3135906 21799676

[B12] Kim WH Song HO Choi HJ Bang HI Choi DY Park H Ethyl gallate induces apoptosis of HL-60 cells by promoting the expression of caspases-8,-9,-3, apoptosis-inducing factor and endonuclease G Int J Mol Sci 2012 13 11912 11922 23109891 10.3390/ijms130911912 PMC3472783

[B13] Gotes J Kasian K Jacobs H Cheng ZQ Mink SN Benefits of ethyl gallate versus norepinephrine in the treatment of cardiovascular collapse in *Pseudomonas aeruginosa* septic shock in dogs Crit Care Med 2012 40 560 572 22020237 10.1097/CCM.0b013e318232d8a6

[B14] Gao S Zhan Q Li J Yang Q Li X Chen W Sun L LC-MS/MS method for simultaneous determination of ethyl gallate and its major metabolite in rat plasma Biomed Chromatogr 2010 24 472 478 19688816 10.1002/bmc.1314

[B15] Wang BS Lin SS Hsiao WC Fan JJ Fuh LF Duh PD Protective effects of aqueous extract of Welsh onion green leaves on oxidative damage of reactive oxygen and nitrogen species Food Chem 2006 98 149 157

[B16] LaemmLi UK Cleavage of structural proteins during the assembly of the head of bacteriophage T4 Nature 1970 227 680 685 5432063 10.1038/227680a0

[B17] Lee JC Kim HR Kim J Jang YS Antioxidant property of an ethanol extract of the stem of *Opuntia ficus-indica* var. Saboten J Agri Food Chem 2002 50 6490 6496 10.1021/jf020388c 12381138

[B18] Ghosh P Devi PG Priya R Amrita A Sivaramakrishna A Babu S Siva R Spectroscopic and in silico evaluation of interaction of DNA with six anthraquinone derivatives Appl Biochem Biotechnol 2013 170 1127 1137 23645388 10.1007/s12010-013-0259-2

[B19] Prophet EB, Mills B, Arrington JB, Sobin LH Armed Forces Institute of Pathology: laboratory methods in Histotechnology 1992 Washington DC: American Registry of Pathology 25 53

[B20] Sharma P Shankar S Agarwal A Singh R Variation in serum lipids and liver function markers in lindane exposed female wistar rats: attenuating effect of curcumin, vitamin C and vitamin E Asian J Exp Biol Sci 2010 1 440 444

[B21] Prakash D Singh BN Upadhyay G Antioxidant and free radical scavenging activities of phenols from onion Food Chem 2007 102 1389 1393 10.1080/09637480601093269 17415953

[B22] Singh BN Singh BR Singh RL Prakash D Sarma BK Singh HB Antioxidant and anti-quorum sensing activities of green pod of *Acacia nilotica* L Food Chem Toxicol 2009 47 778 786 19168114 10.1016/j.fct.2009.01.009

[B23] Teel RW Ellagic acid binding to DNA as a possible mechanism for its antimutagenic and anticarcinogenic action Can Lett 1986 30 329 336 10.1016/0304-3835(86)90058-3 3697951

[B24] Boubaker J Mansour HB Ghedira K Ghedira LC Polar extracts from (Tunisian) *Acacia salicina* Lindl. Study of the antimicrobial and antigenotoxic activities BMC Complemen Altern Med 2012 12 37 10.1186/1472-6882-12-37 PMC3353866 22490278

[B25] Ghomi M Letellier R Liquier J Taillandier E Interpretation of DNA vibrational spectra by normal coordinate analysis Int J Biochem 1990 22 691 699 2205520 10.1016/0020-711x(90)90003-l

[B26] Tyagi G Jangir DK Singh P Mehrotra R DNA interaction studies of an anticancer plant alkaloid, vincristine, using fourier transform infrared spectroscopy DNA and Cell Biol 2010 29 693 699 20662555 10.1089/dna.2010.1035

[B27] Dedon PC Determination of binding mode: intercalation Curr Protoc Nucleic Acid Chem 2001 8 1 13 18428878 10.1002/0471142700.nc0801s00

[B28] Kanakis CD Tarantilis PD Pappas C Bariyanga J Tajmir-Riahi HA Polissiou MG An overview of structural features of DNA and RNA complexes with saffron compounds: models and antioxidant activity J Photochem Photobiol 2009 95 204 212 10.1016/j.jphotobiol.2009.03.006 19395270

[B29] Clarke EGC Clarke ML Cassel , Collier Veterinary Toxicology 1997 London: Macmillan publishers 268 277

[B30] Wakte PS Sachin BS Patil AA Shinde DB Hepatoprotective activity of *Acacia nilotica* flowers Med Chem Drug Discovery 2012 3 152 159

[B31] Guta M Urga K Assefa A Lemma H Addis G Gemeda N Yirsaw k Mudi K Melaku D Antibacterial and acute toxicity study of *Acacia nilotica* Ethiop J Biol Sci 2007 6 43 49

[B32] El-Hadiyah TM Abdulhadi NH Badico EEM Mohammed EYG Toxic potential of ethanolic extract of *Acacia nilotica* (Garad) in rats Sudan J Med Sci 2011 6 1 6

[B33] Raza M Al-Shabanah OA El-Hadiyah TM Al-Majed AA Effect of prolonged vigabatrin treatment on haematological and biochemical parameters in plasma, liver and kidney of Swiss albino mice Sci Pharm 2002 70 135 145

[B34] Teo S Stirling D Thomas S Hoberman A Kiorpes A Khetani V A 90-day oral gavage toxicity study of d-methylphenidate and d I-methylphenidate in Sprague-Dawley rats Toxicol 2002 179 183 196 10.1016/s0300-483x(02)00338-4 12270592

[B35] Al-Mustafa ZH Dafallah AA A study on the toxicology of *Acacia nilotica* Am J Chin Med 2000 28 123 10794124 10.1142/S0192415X00000155

[B36] Rodostits OM Gay CC Blood DC Hinchcliff KW Clinica Veterinaria 2002 9 Rio de Janeiro, RJ, Brazil: Guanabara Koogan, Rio de Janeiro 1737

[B37] Wolford ST Schroer RA Gohs FX Gallo PP Brodeck M Falk HB Ruhren R Reference range data base for serum chemistry and hematology values in laboratory animals J Toxicol Environ Health 1986 18 161 188 3712484 10.1080/15287398609530859

[B38] Johnson-Delaney C Hudelson KS Small mammals Exotic animal companion medicine handbook for veterinarians 1996 Florida: Zoological Education Network 1 62

[B39] Asad M Munir TA Afzal N *Acacia nilotica* leaf extract and glyburide: comparison of fasting blood glucose, serum insulin, beta-thromboglobulin levels and platelet aggregation in streptozotocin induced diabetic rats J Pak Med Assoc 2011 61 247 251 21465938

[B40] Corns CM Herbal remedies and clinical biochemistry Annals of Clin Biochem 2003 40 489 507 10.1258/000456303322326407 14503986

[B41] Hilaly J El Israili ZH Lyoussi B Acute and chronic toxicological studies of *Ajuga iva* in experimental animals J Ethnopharmacol 2004 91 43 50 15036466 10.1016/j.jep.2003.11.009

[B42] Isnard BC Deray G Baumelou M Le Quintree M Vanherweghem JL Herbs and the kidney Am J Kidney Disease 2004 44 1 11 10.1053/j.ajkd.2004.02.009 15211432

[B43] Saad B Azaizeh H Abu-Hijleh G Said S Safety of traditional Arab herbal medicine Evid Based Complement Alternat Med 2006 3 433 439 17173106 10.1093/ecam/nel058 PMC1697757

[B44] Tolman KG Rej R Burtis CA, Ashwood ER Liver function Tietz Textbook of clinical chemistry 1999 3 Philadelphia Pennsylvania: Saunders Company 1125 1177

[B45] Mitruka BM Rawnsley HM Mitruka BM, Rawnsley HM Clinical, Biochemical and Hematology reference values in normal and experimental animals Clinical, biochemical and hematological reference values in normal experimental animals 1981 2 USA: Masson publishing 134 135

[B46] Kannan N Sakthivel KM Guruvayoorappan C Protective effect of *Acacia nilotica* (L.) against acetaminophen-induced hepatocellular damage in wistar rats Adv Pharmacol Sci 2013 2013 1 9 10.1155/2013/987692 PMC3707210 23864853

